# Validating Sentinel Foods in the Diet Quality Questionnaire: Insights from Two Chilean Cohorts of Pregnant Women and Children

**DOI:** 10.3390/nu17182980

**Published:** 2025-09-17

**Authors:** Angela Martínez-Arroyo, Giannella Barisione, Marcela Vizcarra, Natalia Rebolledo, María Luisa Garmendia

**Affiliations:** 1 Centro de Investigación del Comportamiento Alimentario (CEIC), Facultad de Farmacia, Escuela de Nutrición y Dietética, Universidad de Valparaíso, Valparaíso 2381850, Chile; angela.martinez@uv.cl (A.M.-A.); giannella.barisione@gmail.com (G.B.); marcela.vizcarra@uv.cl (M.V.); 2 Center for Research in Food Environments and Prevention of Nutrition-Related Chronic Diseases (CIAPEC), Institute of Nutrition and Food Technology (INTA), University of Chile, Santiago 7830490, Chile; natalia.rebolledo@inta.uchile.cl

**Keywords:** Diet Quality Questionnaire, food consumption, diet quality, pregnant women, children

## Abstract

**Background:** New tools for monitoring diets, such as the Diet Quality Questionnaire (DQQ), may help reduce the costs and burden associated with traditional methods of diet assessment. However, its proposed sentinel foods require validation in target populations. This study aimed to validate the Chilean sentinel foods and describe the performance of DQQ, as well as its Global Dietary Recommendation (GDR) indicators, in two Chilean cohorts. **Methods:** We analyzed dietary data from 1418 pregnant women and 799 children using 24 h recalls. Foods and beverages were classified and ranked into 29 DQQ food groups. Food items that accounted for more than 95% of the total consumption within each of the 29 food groups were selected and identified as sentinels. We estimated the proportion of consumers in each food group and calculated the indicators, as well as their relationship with the consumption of ultra-processed foods (UPFs). **Results:** The pregnant women had a mean age of 29.1 (SD 6.6), and the children had a mean age of 6.2 (SD 0.5). The sentinel foods of six groups (grain-based sweets, other sweets, salty snacks, deep-fried food, and sweet tea/coffee/cocoa) captured under 95% of the group’s total consumption. The pregnant women had a higher frequency of consumption of staple and healthy foods, and the mean GDR score was 9.3 (SD 2.20). The children had higher consumption of unhealthy food groups, and the mean GDR score was 8.1 (SD 2.05). The GDR-protect scores showed a moderate but statistically significant negative correlation with ultra-processed food consumption (*p*-value < 0.0001). **Conclusions:** The DQQ is a suitable tool for collecting dietary data to estimate diet quality using food group-based indicators. Additionally, it is possible to identify different dietary patterns at a crucial stage of life, such as childhood and pregnancy. However, it requires some adaptations of sentinel foods and further testing on other populations before it can be implemented to monitor Chilean diets.

## 1. Introduction

The monitoring of diets should be a global priority because suboptimal diets (e.g., low dietary diversity of staple foods and high consumption of ultra-processed foods (UPFs)) are the leading risk factor for non-communicable diseases (NCDs), which contributed to 11 million deaths and 255 disability-adjusted life-years worldwide in 2017 [1]. Additionally, the production and selection of certain foods for human consumption play a key role in food systems and their impact on our planet [2]. Monitoring diet quality enables the identification of gaps and inequalities in access to nutritious foods, informing public health strategies to improve quality of life, promote health equity among diverse population groups, and foster sustainable food systems [3].

Countries face several challenges in monitoring diet characteristics at the global, regional, national, and subnational scales in order to evaluate and inform nutrition health policies [4]. Less than two-thirds of WHO European Region countries have conducted nationally representative diet surveys in recent decades [5]. In North America, the National Health and Nutrition Examination Survey (NHANES) in the United States, the Canadian Community Health Survey (CCHS), and the National Survey of Health and Nutrition (ENSANUT) in Mexico are conducted systematically, and they are essential resources for monitoring food and nutrition policies [6,7,8]. On the other hand, diet monitoring in South American countries is scarce due to its high cost and the need for specialized infrastructure, trained personnel, and substantial budgets [9,10]. In Chile, the last nationally representative food survey was conducted in 2010. Since then, changes in dietary patterns could have impacted food availability and affordability, as well as individual food consumption preferences, due to the influence of rapidly developing agro-food practices, technology, and economic factors [11]. Therefore, to address these challenges, countries require tools that are standardized, easy to apply and interpret, rapid, and feasible, allowing for comparisons between countries and different time periods. Such tools should also characterize diets in urban and rural settings, across different age and gender groups, and provide a population-level snapshot of diet quality [12].

The Diet Quality Questionnaire (DQQ) is an internationally standardized, fast, and low-burden tool that can be used to monitor population dietary data through a closed-ended list of 29 food groups. These food groups have been associated with nutrition, health, sustainability, and national food-based dietary guidelines [13,14,15]. The DQQ enables the estimation of several indicators of diet quality, such as the Minimum Diet Diversity for Women (MDD-W), Dietary Diversity Score (DDS), and Global Dietary Recommendations (GDR) scores, among others, allowing for the monitoring of dietary quality across the life cycle [16]. The DQQ was previously adapted for use in 140 countries, including Chile, to measure and monitor diet quality [14]. In each country, sentinel foods were identified through interviews with local nutrition experts. However, these sentinel foods still require validation to ensure that they accurately capture dietary intake in different settings and contexts.

In several countries, including China, Ethiopia, Vietnam, Kenya, Colombia, Brazil, and the United States, sentinel food lists have been validated using national or subnational food surveys. These validations have been helpful for confirming or refining the food lists to improve the questionnaire [17,18,19,20]. However, it is unclear whether the sentinel food lists in Chile are capturing the intended food groups. Dietary patterns are dynamic and influenced by a complex interaction of social, cultural, and economic factors; therefore, the DQQ should be validated with specific dietary information [14]. Although the DQQ was designed to monitor diets in adults over 15 years of age, some countries have used this tool to monitor diets in other groups [12,16,21,22]. Thus, our primary objective was to validate sentinel foods in two Chilean cohort studies (pregnant women and school-aged children) using quantitative 24 h recall (24HR) as the reference method. Given that children and pregnant women exhibit distinct dietary preferences and behaviors, we aimed to identify sentinel foods and propose improvements to the questionnaire as well as indicators of diet quality for the two cohorts. Additionally, we examined the association between the GDR indicator and its NCD risk and protect subcomponents, DDS scores, and UPF consumption considering that these factors have been associated with poorer diet quality.

## 2. Materials and Methods

### 2.1. Participants

We used dietary data from two Chilean cohort studies conducted at the Center for Research in Food Environments and Prevention of Nutrition-Related Chronic Diseases (CIAPEC-INTA). The first cohort was the Food and Environment Chilean Cohort (FECHIC), initiated in 2016, which enrolled 962 preschoolers from low- and middle-income families residing in the urban area of Southeast Santiago. Briefly, this cohort study was established to assess changes in dietary intake before and after the implementation of the Chilean Law of Food Labelling and Advertising [23]. The participants were recruited at ages 3–6 years. The inclusion criteria were mothers responsible for food purchases at home and childcare, without a history of mental illness, and children without gastrointestinal diseases that would affect their habitual food consumption, as well as singleton births. Details on the participant selection procedure and the study protocol have been described elsewhere [24].

The second cohort was the Chilean Maternal and Infant Cohort Study II (CHiMINCS-II), which started in 2020 and included 1954 pregnant women residing in Puente Alto County. The inclusion criteria were as follows: (1) age > 18 y, (2) <15 weeks of gestation at the first prenatal visit, and (3) no intention to move outside of Santiago within the next 2 years. Women were excluded if they had a high-risk pregnancy (e.g., preeclampsia or pre-existing diabetes). The details of the study protocol have been described elsewhere [25].

For this analysis, we included all FECHIC children with a single dietary assessment between February and September 2018 (*n* = 801). Two children were excluded, one due to self-reported unusual consumption and implausible energy intake (<140 kcal/day) and the other due to missing weight status. From the CHiMINCS-II study, we included pregnant women with a single dietary data assessment between October 2020 and February 2022 (*n* = 1418). We used dietary data collected during the second trimester to minimize changes in diet related to symptoms associated with pregnancy (e.g., vomiting and nausea).

In both cohorts, participants with a single 24 h recall (24HR) were considered, and sociodemographic data and anthropometric measurements obtained during the same clinic visit or by telephone were analyzed to characterize the sample.

### 2.2. Dietary Data Collection

Dietary intake was assessed through a single 24HR collected by trained dietitians using the software SER24H v 1.0 (CIAPEC, Santiago, Chile). This software is based on a multiple-step methodology [26] and includes the most common foods and culture-based recipes of dishes consumed in Chile. SER24H generates outputs of food and beverage intake in grams, which are linked to nutritional composition databases. Additionally, the post-processing SER24H databases enable the classification of foods and beverages using the NOVA system [27]. Details on SER24H have been published previously [28].

During the interview (conducted face-to-face for children or by telephone for pregnant women), dietitians used a Photographic Atlas of common Chilean foods and beverages to help the participants estimate the size of the portions they had consumed [29]. The dietitians also requested information about the brand names and characteristics of each packaged food item consumed in the previous 24 h.

The pregnant women reported their own intake, and while the children were accompanied by one caretaker (a parent or a guardian) who was aware of the child’s intake during the previous day.

### 2.3. Diet Quality Questionnaire (DQQ)

Briefly, the DQQ is an easy and fast tool to apply and uses binary questions (yes/no) regarding 29 food groups consumed the previous day or night [14]. The food groups included in this tool were (1) foods made from grains; (2) whole grains; (3) white roots, tubers, and plantains; (4) legumes; (5) vitamin A-rich orange vegetables; (6) dark green leafy vegetables; (7) other vegetables; (8) vitamin A-rich fruits; (9) citrus; (10) other fruits; (11) baked/grain-based sweets; (12) other sweets; (13) eggs; (14) cheese; (15) yogurt; (16) processed meats; (17) unprocessed red meat (ruminant); (18) unprocessed red meat (non-ruminant); (19) poultry; (20) fish and seafood; (21) nuts and seeds; (22) packaged ultra-processed salty snacks; (23) instant noodles; (24) deep fried foods; (25) fluid milk; (26) sugar-sweetened beverages (soft drinks); (27) fruit juice and fruit-flavored drinks; (28) sweet tea/coffee/cacao; and (29) fast food. DQQ development was aligned with global recommendations for micronutrient adequacy, adherence to healthy diets, and dietary risk factors for NCDs, including the consumption of ultra-processed foods [16]. Each food group is represented by 2–7 sentinel foods. Through an interview, key informants and nutrition experts fluent in the native language identified specific sentinel foods (i.e., the most commonly consumed items that capture most of the 29 food groups) belonging to each food group and the names by which the population best understands them [19]. The Chilean DQQ can be found in Supplementary File S1.

### 2.4. Classification and Validation of DQQ’s Sentinel Foods

To validate the sentinel foods, we selected all foods and beverages consumed in amounts over 15 g from the 24HRs. For mixed dishes, the main ingredients that comprised each dish were disaggregated and classified. For example, the traditional Chilean dish *“cazuela de pollo”* was classified into the groups 3, 5, and 15 because its main components (e.g., potatoes, chicken, zapallo squash, and carrots) each exceeded the 15 g threshold and contributed almost 70% of the dish [30].

We reviewed the data in three steps: (i) all foods and beverages reported in the 24HRs were classified into the 29 DQQ groups and ranked according to their contribution to the consumption frequency of each group following the Food Group Classification Guide and the Minimum Dietary Diversity Guide [31]; (ii) we identified only the sentinel foods and beverages described in the current Chilean DQQ; and (iii) we identified some foods and beverages that could improve the frequency of contribution of sentinel foods in the Chilean DQQ, which would improve the capture rate of sentinel foods above a 95% threshold [16].

### 2.5. Diet Quality Indicators:

Through the DQQ food groups, we estimated several diet quality indicators, such as Minimum Dietary Diversity for Women of Reproductive Age (MDD-W), Dietary Diversity Score (DDS), and Global Dietary Recommendations (GDR) scores, including the NCD-Protect and NCD-Risk subcomponents (indicators of protective or risk dietary factors for NCDs). The overall GDR score ranges from 0 to 18, and it is composed of two subcomponents, each rated from 0 to 9 points. The NCD-Protect Score indicates recommendations for “healthy” foods such as fruits, vegetables, legumes, nuts, seeds, whole grains, and dietary fiber. The NCD-Risk Score provides recommendations for limiting specific dietary components, including total fat, saturated fat, sodium, free sugars, processed meat, and unprocessed red meat, which could also serve as a proxy for UPF intake. A lower overall GDR score and NCD-Protect Score and a higher NCD-Risk Score indicate poorer diet quality [16]. The GDR score was calculated as follows: NCD-Protect − NCD-Risk + 9 = GDR score [32]. We estimated all indicators using the original sentinel foods of the DQQ for children and pregnant women.

We included other indicators to evaluate diet quality such as (i) zero vegetable or fruit consumption; (ii) Protective Food Consumption (proportion of the population that consumed at least one fruit, at least one vegetable, and at least one whole grain, legume, nut, or seed); (iii) more than one sugary food or beverage (proportion of the population that consumed more than one sugary food or beverage in the previous day or night); and (iv) more than one salty ultra-processed food (proportion of the population that consumed more than one salty food in the previous day or night). Details of the development of these indicators are available in the Indicator Guide of the Diet Quality Project [32]. Information on how the indicators in this study were calculated from the DQQ food groups is presented in Supplementary File S2.

### 2.6. Ultra-Processed Food Consumption

All food and beverages reported from the 24HRs were categorized according to the NOVA classification (Group 1: natural food or minimally processed foods; Group 2: culinary ingredients; Group 3: processed foods; and Group 4: UPFs) [33]. We estimated the energy intake of each NOVA group and estimated the energy contribution of each group to the total daily energy intake for each participant.

### 2.7. Anthropometric Data

Trained dietitians collected anthropometric measures during follow-up visits for both cohorts using standardized procedures. Height was measured using a portable stadiometer (SECA 222, to the nearest 0.1 cm), and weight was measured using a digital electronic scale (TANITA BC-418, to the nearest 0.1 Kg). All instruments were calibrated twice a month. Weight and height measurements were taken in duplicate, and the mean of the two measurements was used in this research. For the children, age- and sex-specific body mass index (BMI) z-scores were calculated, and the participants were categorized based on their weight status using the World Health Organization (WHO) Growth Reference 2007. We defined the weight status as underweight (≤−1 Standard Deviation (SD)), normal weight (<−1 SD and +1 SD), overweight (≥+1 SD and <+2SD), and obesity (≥+2SD) [34].

For the pregnant women, we had access to maternal weight measurements taken at each trimester and delivery from clinical records. For this study, we used the weight and height from an electronic clinical visit to calculate the pre-pregnancy body mass index (BMI). The pre-pregnancy BMI was used to classify the pregnant women as underweight, normal weight, overweight, and obesity based on the WHO criteria.

### 2.8. Other Variables

During clinic visits, the pregnant women and parents or caregivers of the children provided sociodemographic characteristics using a semi-structured questionnaire. The main variables included in this study were (i) participant age (years); (ii) participant sex (self-reported by the children, categorized as female or male); maternal educational level (mother of the children and pregnant women, self-reported, categorized as ≤12 years (high school complete or incomplete) or >12 years (college or higher)), and day (weekday yes/no) and season (autumn, winter, spring, or summer) when the 24HR was collected.

### 2.9. Statistical Analysis

We calculated the relative and cumulative frequencies of each type of food and beverage within each of the 29 DQQ food groups, ranking them in descending order. We identified foods and beverages that accounted ≥ 95% of the group consumption, as reported in prior studies [18,20,21].

We estimated the prevalence of consumption for each of the 29 DQQ food groups according to the original sentinel Chilean foods and beverages. We estimated the indicators separately for the children and pregnant women [16,32].

To evaluate the performance of DQQ indicators, we correlated the percentage of calories from UPF consumption with GDR, DDS, NCD-Risk, and NCD-Protect scores using the Spearman test. Two-sided *p*-values < 0.05 were considered statistically significant. All analyses were conducted using Stata 18 (StataCorp, College Station, TX, USA).

## 3. Results

### 3.1. Characteristics of the Participants

The sociodemographic and anthropometric characteristics of both cohorts are presented in Table 1. The mean age of the FECHIC participants was 6.2 years (SD 0.53), and 51.3% of the participants were girls. Based on the BMI z-scores, approximately half of the children were affected by excess weight or obesity, and 59.1% of their mothers had completed at least 12 years of education. Regarding dietary data, 14.4% of the 24HRs were collected on weekends, and most were during the autumn and winter seasons, which are the cold seasons in Chile. Almost half (48.4%) of the daily energy intake was provided by UPFs. In the CHiMINCs II cohort, the pregnant women were 29.1 years (SD 6.6), and 68.3% had completed 12 years of education. There was a high prevalence of overweight in pregnant women, with three-quarters of women affected by pre-gestational overweight or obesity. The dietary data were mostly collected during spring and summer (55.5% of 24HRs), and 15.6% of the 24HRs were collected on weekends. The relative contribution of UPF consumption was about one-third of the daily energy intake.

### 3.2. Food Group Classification

Foods and beverages reported in the 24HRs, categorized according to the Food Group Classification Guide, are shown in Table 2. A total of 20,152 foods and beverages were reported by the pregnant women and 10,778 by the children. The foods in each food group are presented in the order specified by the Chilean DQQ. The ranking columns show the frequency of consumption of the foods and beverages in each cohort. For example, sentinel food items such as bread, pasta, and rice accounted for 96% of the grain consumption among those who consumed grains. Overall, the sentinel foods performed well: in 23 of the 29 food groups, they captured at least 95% of the items reported. However, six groups (Groups 11, 12, 16, 22, 24, and 26) were below this threshold, indicating a need for improvement. Interestingly, all of these were unhealthy food groups. We identified some non-sentinel foods and beverages from the unhealthy groups that were poorly captured. For example, cereal bars were classified into the grain-based sweet group (Group 11), with a frequency of consumption of 4.1% in the pregnant women and 11.7% in the children. Other non-sentinel foods with high frequencies were jellies and milk-based desserts (Group 12); pate/spread (Group 16); saltine crackers (Group 22); and fried chicken (Group 24). The sentinel foods in Group 26 showed low frequencies because the classification guideline indicates that flavored milk or milk with cocoa should be classified into Groups 25 and 26. However, the Chilean DQQ did not consider those foods. More details about the identified improvements to the DQQ are presented in Supplementary File S1.

### 3.3. Frequency of Sentinel Food Group Consumption

**Figure 1:**
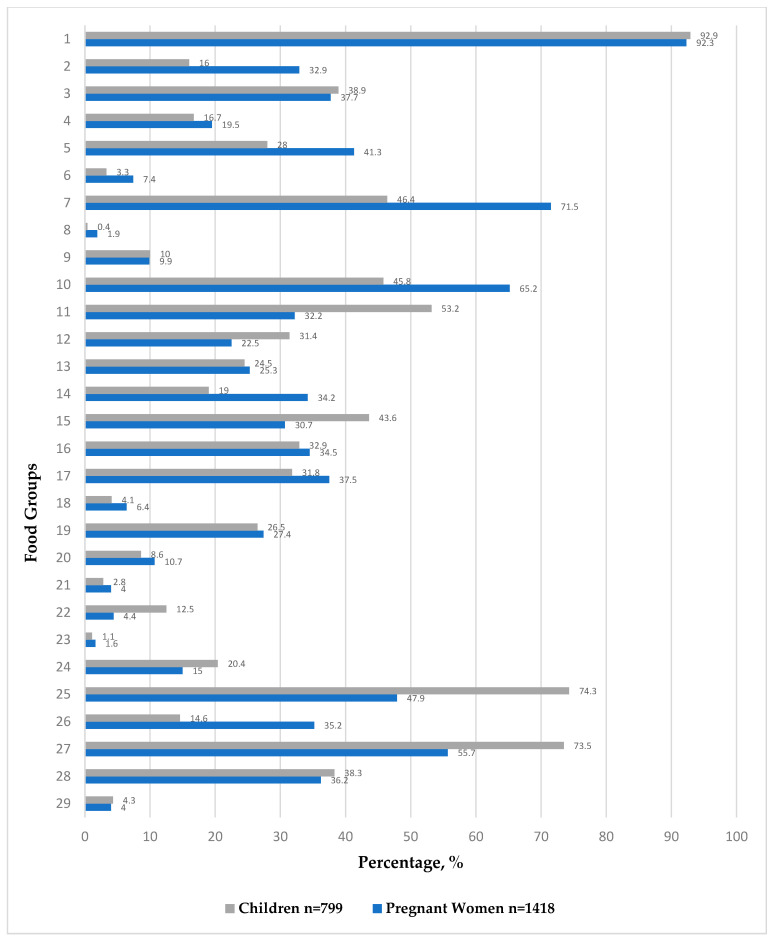
Percentage of pregnant women and children consuming each food group in Chilean DQQ, according to reports from 24HR. Chile DQQ Group 1: staple foods made from grains; Group 2: whole grains; Group 3: white roots, tubers, and plantains; Group 4: legumes; Group 5: vitamin A-rich orange vegetables; Group 6: dark green leafy vegetables; Group 7: other vegetables; Group 8: vitamin A-rich fruits; Group 9: citrus; Group 10: other fruits; Group 11: baked/grain-based sweets; Group 12: other sweets; Group 13: eggs; Group 14: cheese; Group 15: yogurt; Group 16: processed meats; Group 17: unprocessed red meat (ruminant); Group 18: unprocessed red meat (non-ruminant); Group 19: poultry; Group 20: fish and seafood; Group 21: nuts and seeds; Group 22: packaged ultra-processed salty snacks; Group 23: instant noodles; Group 24: deep fried foods; Group 25: fluid milk; Group 26: sweet tea/coffee/cocoa; Group 27: fruit juice and fruit-flavored drinks; Group 28: soft drinks; Group 29: fast food.

The percentage of consumption of each DQQ food group in the CHiMiNCs-II and FECHIC cohorts are shown in Figure 1. The most frequently consumed food group was foods made from grains such as white bread, pasta, or rice (~93%), while the food groups with lower frequencies of consumption in both Chilean cohorts were vitamin A-rich fruits (~1.15%), instant noodles (~1.35%), and nuts and seeds (~3.4%). Additionally, we observed consumption patterns that differed between the cohorts. The pregnant women showed a higher consumption of healthy food groups, such as whole grains (32.9%), vitamin A-rich orange vegetables (41.3%), other vegetables (71.5%), and other fruits (65.2%). On the other hand, the children showed a higher consumption of milk (74.3%) and unhealthy food groups, such as grain-based sweets (53.2%), other sweets (31.4%), packaged ultra-processed salty snacks (12.5%), deep-fried foods (20.4%), and fruit juice and fruit-flavored drinks (73.5%).

### 3.4. DQQ Indicators

The scores of the pregnant women and children for the DQQ indicators are shown in Table 3. Specifically, the pregnant women had higher GDR, NCD-Protect, and DDS scores and a lower NCD-Risk score than children. Two-thirds of the pregnant women achieved the MDD-W recommendation (score ≥ 5). In contrast, the children had worse outcomes for the indicators that reflect unhealthy diets: 23% of the children did not consume any vegetables or fruits, and they had a higher proportion of consumption of sugary foods or beverages, as well as salty, ultra-processed foods.

### 3.5. Correlations Between Diet Quality Scores and NOVA Classification

The correlations between diet quality scores, the consumption of NOVA food groups, and total energy intake are presented in Table 4. We found that total energy intake was negatively and weakly correlated with the GDR score in both cohorts (*p*-value < 0.001). When we observed the indicators, the GDR score was negatively correlated with the percentage of energy from UPFs (−0.31 in pregnant women and −0.39 in children). In contrast, the GDR score was positively correlated with the percentage of energy from unprocessed or minimally processed foods (0.39 in pregnant women and 0.37 in children). The indicator NCD-protect was positively correlated with the percentage of energy from unprocessed or minimally processed foods (0.27 in pregnant women and 0.29 in children), and NCD-risk was positively correlated with the percentage of energy from UPFs (0.33 in pregnant women and 0.33 in children). The DDS showed a positive correlation with total energy intake and the percentage of energy from unprocessed and minimally processed foods, but it was negatively correlated with the percentage of energy from UPFs (*p*-value < 0.001).

## 4. Discussion

Our study validated the sentinel foods of the Chilean DQQ using dietary data from two key populations for public health policies: children and pregnant women. We found that 23 of the 29 food groups achieved nearly 95% coverage of all the foods and beverages consumed, suggesting that the DQQ could be a valid tool for monitoring diet in Chilean children and pregnant women from urban settings. However, some sentinel foods of the Chilean DQQ, mainly those from unhealthy food groups, were poorly captured by the DQQ. Notably, the DQQ prevalence of food consumption and indicators allowed for the monitoring of the dietary quality of pregnant women and children. Additionally, we observed that DQQ indicators exhibited the expected correlation (i.e., GDR had a positive correlation with the percent of energy from minimally processed foods and a negative correlation with UPF intake).

We found that some widely consumed foods were not included as sentinels in the Chilean DQQ. In Group 11 (grain-based sweets), churros showed a low consumption (<1%), while cereal bars were frequently consumed (~4% to ~12%). In the Chilean context, cereal bars are a highly consumed snack among children and adolescents; they are similar to cookies and contribute free sugars to the diet [35,36]. For Group 12 (other sweets), the current sentinel foods accounted for ~72% of the reported items in both cohorts. However, jellies and industrialized milk-based desserts were frequently consumed in both cohorts (~24%) but they were not considered as sentinels in the DQQ adaptation [14]. For Group 16, we found that bacon was not frequently consumed (<1%) in both cohorts, while pate/spread had a higher frequency of consumption (~10% among pregnant women and ~2% in children). Since bacon is not culturally relevant in Chile like in other countries, pate/spread would be a more appropriate sentinel food. For Group 22 (packaged ultra-processed salty snacks), saltine crackers (an ultra-processed item considered like chips) were frequently consumed by pregnant women and children. However, they are not included as sentinel foods in any of the global DQQ instruments based on their global exclusion criteria. In Group 24 (deep-fried foods), we propose removing “wontons” because they are not commonly consumed (<1%) and replacing “nuggets de pollo” with the broader term “nuggets” to capture both fish and chicken nuggets. We also suggest adding “fried chicken” because it was a commonly consumed item. Lastly, we found that flavored milk, milkshakes, and cocoa powder in milk (e.g., Nesquik or Milo brands) were commonly consumed (43% of pregnant women and 84% of children). However, these items were not considered as sentinel foods in the Chilean DQQ, and we found that other countries include them in Group 26. The aim of Group 26 is to capture beverages with added sugar. Nowadays, sweetened flavored milk and cocoa powder are often produced with non-caloric sweeteners following the implementation of Chile’s labeling law, which has prompted the industry to reformulate these products to be low in sugar [37]. Thus, the DQQ question should be reformulated to include only flavored milk, milkshakes, and cocoa powder with added sugars. If they were included as sentinel foods, it would improve the capture rate in Groups 25 and 26. Further studies on other samples are needed to corroborate that these are indeed the best sentinel foods.

Our findings demonstrate that the DQQ can be used to monitor populations of different ages. The consumption of healthy food groups by the pregnant women (e.g., whole grains, fruits, and vegetables) was higher than in the children, likely because they are more aware of the importance of nutrition during pregnancy. In contrast, the children had a higher prevalence of consuming unhealthy food groups (e.g., sugary foods and beverages and UPFs), which reflects the influence of the food environments which children are exposed to during childhood.

Comparisons with the DQQ results from the Gallup World Poll (GWP) in Chile in 2021 (sample of adults, *n =* 1000, aged >15 years) revealed that our study had a lower consumption of vitamin A-rich vegetables and fish [38]. The GWP reported that 71% and 23% of their sample consumed vitamin A-rich vegetables and fish, respectively, while in our study, the prevalence was ~35% and ~9.7%, respectively (averaged across both cohorts). These differences could be attributed to variations in methods, portion size cutoffs, and population characteristics. For example, carrots and red bell peppers are consumed in small amounts in mixed dishes. The 15 g cutoff that we applied was important to avoid falsely inflating the prevalence of consumption of this DQQ food group. Regarding fish consumption, our findings are consistent with the 2017 National Health Survey, which reported that 9.2% of Chileans consume fish at least twice a week [39]. Different samples, in terms of age, income (FECHIC and CHiMINCs consist of low- to middle-income and urban participants), setting, education level, year, and methodologies of collection, may explain these differences.

We highlight that the DQQ can capture cultural differences in dietary patterns between countries. For instance, the consumption of pulses and beans in Chile is low at ~18% for both cohorts compared with Brazil, which consumes beans daily (79%) as a staple food [19]. The consumption of food from a fast-food restaurant in the US is more frequent at 34% compared to Chile (~4%) and Brazil (3%) [19]. This finding is crucial because the DQQ was designed to monitor global diets across different contexts [14].

The DQQ indicators also captured differences in the prevalence of food group consumption between the cohorts. The GDR score of the pregnant women (9.3 points) was similar Chilean adults in the GWP (9.9 points) [38], but lower than the scores found in low- and middle-income countries (LMICs), which range from 10 to 11 points [17]. Subcomponents of the GDR, such as NCD-Protect and Risk, also presented lower scores in our cohorts compared to adults in Chile but were comparable to those observed in LMICs [38]. Additionally, 7% of the pregnant women and 23% of the children did not consume any vegetables or fruits on the day of the survey, indicating a possible food insecurity. Finally, the pregnant women had a higher Protective Food Consumption indicator score. They are likely more aware of health because they might receive nutritional counseling in public health centers. In contrast, the children had a higher proportion of unhealthy food consumption, reflecting a low GDR and higher NCD-Risk scores, indicating poor dietary quality. As children grow, they become more exposed to social acceptance of peers and obesogenic food environments promote unhealthy food consumption [40,41]. Our cohorts represent families from low- to middle-income backgrounds. Chile still requires policy efforts, such as controlling the sale of unhealthy food outside schools and providing monetary subsidies, to increase the consumption of healthy food among low-income families [42].

The MDD-W and DDS are well-established predictors of micronutrient adequacy among reproductive and pregnant women in LMICs [22,31], but they have also been used in children and adolescents in upper-middle- and high-income countries [43,44,45]. In our study, two-thirds of the pregnant women and almost half of the children met the recommendation, with mean scores of 5.0 and 4.4, respectively. Similar findings were reported from two cross-sectional quantitative dietary intake surveys conducted in China (mean score: 4.7) and Mexico (mean score: 4.3) [45]. A multicenter Latin American study found that the mean DDS was 4.7, and 57.7% of women achieved a minimum diverse diet [46]. LMICs showed slightly lower values [22]. As previously mentioned, the collection of 24HRs occurred in different seasons (pregnant women in spring–summer and children in autumn–winter); therefore, the availability and purchase of fruit and vegetables (the leading group for the MDD) can impact consumption and the MDD indicator [47]. Although dietary diversity is not a sufficient indicator of overall diet quality, these indicators can be used to evaluate the impact of programs, inform policy decisions, and set targets [31]. In 2025, the MDD was formally adopted by the Food and Agriculture Organization and the United Nations Statistical Commission (56th session) for monitoring and tracking progress in achieving Sustainable Development Goal 2, which focuses on ending hunger, achieving food security, and improving nutrition, as well as promoting sustainable agriculture [16].

Our findings for the consumption of the NOVA classification groups were in the expected direction. Although the grade showed a moderate correlation, the GDR score was positively correlated with natural and minimally processed foods and negatively correlated with UPFs. Additionally, the subcomponent NCD-Protect was negatively correlated to UPFs, while NCD-Risk was positively associated with UPFs, which are consistent with the findings observed in the US and Brazil [19]. On the other hand, the DDS was better correlated with natural and minimally processed foods given that dietary diversity is associated with an adequate level of micronutrients [43,44]. UPFs accounted for 34% of the total energy intake in the pregnant women and 48% in the children, similar to the values reported in other Chilean studies, confirming the unhealthy diet in Chile [35,48]. Specific categories of UPFs of the DQQ, such as baked/grain-based sweets, other sweets, packaged ultra-processed salty snacks, soft drinks, and fruit drinks, were highly consumed, mainly by the children. Early exposure to ultra-processed foods during pregnancy and childhood can influence food preferences throughout life and impact future health. Promoting the consumption of minimally processed foods and reducing the consumption of ultra-processed foods will improve dietary quality, particularly among vulnerable populations, such as pregnant women and children [49].

This study has both strengths and limitations. One of its key strengths is providing valuable and high-quality quantitative dietary data from two cohorts with different age groups, which enabled the verification of the true consumption of sentinel foods and the proposal of improvements to the tool. This step is essential after adapting the key informants, especially if a food population survey is not available. Although 24HR is recommended to validate sentinel foods, a single 24HR was not sufficient to evaluate some seasonal sentinel foods. For instance, the pregnant women had higher consumption frequencies for fruit and vegetables, probably because their 24HRs were recorded during the hot season (spring and summer) when some fruits (berries, grapes, watermelon, melon, peaches, and plums) and some vegetables are cheaper and more readily available. However, individual preferences, recall bias, and response bias could be present, given the social desirability, because of the high prevalence of overweight and obesity in pregnant women as they take better care of their diet during pregnancy [50]. The retrospective analysis of the DQQ from 24HR data can also affect the response. The 24HRs are open questionnaires conducted by trained dietitians, and the DQQ is based on a closed list of sentinel foods. Future longitudinal assessments should first apply the DQQ and then 24HR on the same day to evaluate the reproducibility and replicability of the DQQ. In addition, we need to validate the DQQ in other populations, because our study sample was relatively homogeneous (urban, low- to middle-income). Also, pregnant women are a unique group in terms of nutritional needs and dietary recommendations; therefore, their requirements and behaviors differ significantly from those of non-pregnant individuals. Our findings are valuable, providing evidence for food consumption and diet quality monitoring in Chile.

## 5. Conclusions

Monitoring dietary patterns is essential for understanding the impact of diet on health and nutrition. Tools like the DQQ, which are easy and fast to use, are crucial for evaluating public health policies in countries where systematic food surveys are not conducted. Our findings suggest that the sentinel foods of the DQQ can capture a high proportion of the Chilean diet. However, the DQQ still needs to be improved by incorporating other foods and tested in different samples, such as adults in other settings in Chile. The DQQ and its indicators may be sensitive to variations in dietary intake between groups and exhibit a moderate correlation with indicators of dietary quality, such as UPF consumption. Subsequent studies should assess the sensitivity of incorporating the proposed sentinel foods and the reliability and the temporal stability of the DQQ before it is used to monitor diets at the national or global level.

## Figures and Tables

**Table 1:** Sociodemographic, nutritional, and dietary characteristics of pregnant women and children.

	Pregnant Women	Children
	Total (*n* = 1418)	Total (*n* = 799)
Age, y ^a^	29.1 (6.6)	6.2 (0.53)
Female, % (*n*)	100 (1418)	51.3 (410)
Weight, (kg) ^a,d^	74.6 (16.13)	25.5 (5.41)
Height, (cm) ^a,d^	158.9 (5.84)	120.1 (5.88)
BMI, (kg/m^2^) ^a,b,d^	29.5 (6.11)	17.6 (2.71)
Weight status		
Underweight, % (*n*)	0.7 (9)	4.0 (32)
Normal weight, % (*n*)	27.7 (328)	45.7 (365)
Overweight, % (*n*)	34.6 (478)	28.8 (230)
Obesity, % (*n*)	41.0 (567)	21.5 (172)
Schooling (y) ^c^		
≤12 years, % (*n*)	68.3 (968)	59.1 (472)
>12 years, % (*n*)	31.7 (450)	40.9 (327)
Season		
Autumn, % (*n*)	26.5 (376)	67.1 (536)
Winter, % (*n*)	17.9 (254)	18.3 (146)
Spring, % (*n*)	20.2 (287)	0.1 (1)
Summer, % (*n*)	35.3 (501)	14.5 (116)
24HR day		
Weekend (yes), % (*n*)	15.6 (221)	14.4 (115)
Total dietary energy (kcal/day) ^a^	1604 (618.59)	1412 (418.57)
NOVA food classification system,%kcal ^a^		
Group 1 ^a^	37.9 (16.34)	31.9 (17.1)
Group 2 ^a^	8.9 (7.12)	7.8 (8.78)
Group 3 ^a^	18.9 (13.82)	11.9 (10.60)
Group 4 ^a^	34.4 (19.41)	48.4 (19.27)

^a^ Values are given as mean (SD) or % (*n*). ^b^ Pre-pregnancy body mass index (BMI). Pre-pregnancy BMI was used to classify the pregnant women as underweight, normal weight, overweight, and obese using the World Health Organization criteria. The children’s weight status was based on BMI for age z-scores (BAZ) using the WHO reference 2007 child growth standards for 5- to 19-year-old children. ^c^ Mandatory education includes basic and secondary education, 12 years in total. For the children, we used the educational level of their mothers. ^d^ Number of missing data points for pregnant women: 31 for weight; 24 for height; and 36 for BMI. BMI, body mass index; 24HR, 24 h recall; NOVA, food classification system: Group 1, unprocessed or minimally processed foods; Group 2, processed culinary ingredients; Group 3, processed foods; Group 4, ultra-processed foods.

**Table 2:** Frequencies of consumption of DQQ’s sentinel foods (>15 g) in pregnant women and children from Chile ^a^.

Sentinel Food	Pregnant Women			Children		
	*n* ^b^	%	Ranking	*n* ^b^	%	Ranking
Staple foods made from grains (Group 1)						
White bread	1900	61.1	1	947	57.2	1
Pasta	397	12.8	3	290	17.5	3
Rice	653	21.0	2	365	22.1	2
Others	160	5.1	-	53	3.2	-
Whole grain (Group 2)						
Oats	104	15.9	3	20	12.4	3
Corn	311	47.6	1	91	56.2	1
Wheat berries	18	2.8	4	4	2.5	4
Whole grain bread	199	30.4	2	32	19.8	2
Quinoa	3	0.5	5	1	0.6	5
Others	19	2.9	-	14	8.6	
White root/tubers (Group 3)						
Potato	583	98.9	1	363	100	1
Others	6	1.1	-	0	0	-
Legumes (Group 4)						
Beans	111	29.7	2	55	35	1
Chickpeas	14	3.7	4	6	3.9	4
Lentils	80	21.4	3	49	32	2
Peas	115	30.8	1	33	21.6	3
Soy meat	9	2.4	5	0	0	5
Hummus	3	0.8	6	0	0	6
Others	42	11.2	-	10	6.5	-
Vitamin A-rich orange vegetables (Group 5)						
Carrots	756	67.1	1	150	56.2	1
Zapallo squash	282	25	2	117	43.8	2
Red peppers	89	7.9	3	0	0	3
Dark green leafy vegetables (Group 6)						
Broccoli	62	43.4	1	13	50	1
Chard	30	21	2	9	34.6	2
Spinach	45	31.5	3	3	11.5	3
Others	6	4.2	-	1	3.9	-
Other vegetables (Group 7)						
Tomatoes	762	37.6	1	241	44.1	1
Lettuce	488	24.1	2	145	26.5	2
Cucumber	81	4	5	31	5.7	4
Green beans	208	10.3	3	30	5.5	5
Cabbage	125	6.2	4	37	6.8	3
Cauliflower	38	1.9	10	6	1.1	8
Zucchini	66	3.3	7	12	2.2	7
Beet	54	2.7	8	6	1.1	9
Celery	72	3.6	6	26	4.8	6
Artichoke	6	0.3	12	4	0.7	10
Asparagus	7	0.3	11	1	0.2	11
Mushrooms	52	2.6	9	1	0.2	12
Others	70	3.5	-	7	1.3	-
Vitamin A-rich fruits (Group 8)						
Cantaloupe	11	36.7	2	1	33.3	2
Apricots	4	13.3	3	0	0	3
Mango	14	46.7	1	2	66.7	1
Loquat	0	0	4	0	0	4
Others	1	3.3	-	0	0	-
Citrus (Group 9)						
Orange	92	57.9	1	55	60.4	1
Mandarin	66	41.5	2	36	39.6	2
Others	1	0.6	-	0	0	-
Other fruits (Group 10)						
Banana	285	15.7	3	118	20.2	2
Apple	288	15.9	2	140	23.9	1
Pear	67	3.7	7	30	5.1	6
Peaches	244	13.5	4	42	7.2	5
Plums	17	0.9	11	2	0.3	11
Kiwi	23	1.3	10	5	0.9	9
Watermelon	67	3.7	8	9	1.5	8
Avocado	427	23.6	1	111	18.9	3
Grapes	100	5.5	5	53	9.1	4
Cherries	24	1.3	9	0	0	13
Strawberries	92	5.1	6	21	3.6	7
Raspberries	5	0.3	13	2	0.3	10
Blackberries	0	0	14	1	0.2	12
Blueberries	12	0.7	12	0	0	14
Others	161	8.9	-	51	8.7	-
Grain-based sweets (Group 11)						
Cookies	266	40.2	1	371	56.1	1
Cakes	133	20.1	2	50	7.6	3
Quick sweet breads	129	19.5	3	106	16	2
Chilean pastries	53	8	4	34	5.1	5
Churros	5	0.8	8	2	0.3	8
Calzones rotos	10	1.5	6	11	1.7	6
Donuts	10	1.5	7	3	0.5	7
Cereal bars ^c^	27	4.1	5	77	11.7	4
Others	29	4.4	-	7	1.1	-
Other sweets (Group 12)						
Candy	21	3	6	18	4.2	5
Chewy candies	60	8.6	4	30	6.9	4
Chocolates	169	24.3	2	78	18.1	3
Ice cream or popsicle	185	26.6	1	166	38.6	1
Manjar	53	7.6	5	12	2.8	6
Jellies and milk-based dessert ^c^	165	24.4	3	102	23.9	2
Others	42	6.0	-	24	5.6	-
Eggs (Group 13)						
Eggs	447	100	1	219	100	1
Cheese (Group 14)						
Hard cheese	495	75.9	1	146	92.4	1
Fresh cheese	157	24.1	2	12	7.6	2
Yogurt (Group 15)						
Yogurt	481	94.5	1	465	98.1	1
Cultured milk	28	5.5	2	9	1.9	2
Processed meats (Group 16)						
Ham	472	60.5	1	158	50.5	1
Bologna	21	2.7	6	5	1.6	5
Hot dogs	128	16.4	2	119	38	2
Chorizo sausage	7	0.9	7	5	1.6	6
Longaniza sausage	33	4.2	5	11	3.5	3
Salami	35	4.5	4	10	3.2	4
Bacon	5	0.6	8	0	0	8
Patés ^c^	77	9.9	3	5	1.6	7
Others	2	0.3	-	0	0	-
Unprocessed red meat (ruminant) (Group 17)						
Beef	598	92.1	1	277	90.8	1
Beef liver	6	0.9	2	0	0	2
Lamb	0	0	3	0	0	3
Goat	0	0	4	0	0	4
Others	45	6.9	-	28	9.2	-
Unprocessed red meat (non-ruminant) (Group 18)						
Pork	96	100	1	34	100	1
Poultry (Group 19)						
Chicken	448	97.2	1	233	93.9	1
Turkey	13	2.8	2	15	6.1	2
Fish and seafood (Group 20)						
Fish	62	32.9	1	25	34.2	1
Jurel	21	11.2	4	24	32.3	2
Tuna	54	28.7	2	23	31.5	3
Sardines	2	1.1	5	1	1.4	4
Seafood	49	26.1	3	0	0	5
Nuts and seeds (Group 21)						
Peanuts	34	32.1	1	14	53.9	1
Peanut butter	6	5.7	4	1	3.9	4
Almonds	34	32.1	2	3	11.5	3
Walnuts	26	24.5	3	7	26.9	2
Chilean hazelnut	1	0.9	5	0	0	5
Chilean pine nuts	0	0	7	0	0	6
Chestnuts	1	0.9	6	0	0	7
Others	4	3.8	-	1	3.9	-
Packaged ultra-processed salty snacks (Group 22)						
Potato chips	50	27.9	2	68	41	1
Ramitas	11	6.2	3	17	10.2	4
Cheetos	5	2.8	6	1	0.6	6
Doritos	7	3.9	5	15	9	5
Suflés	11	6.2	4	21	12.7	3
Saltine crackers ^c^	95	53.1	1	44	26.5	2
Instant noodles (Group 23)						
Instant soup	21	81	1	4	40	2
Instant noodles	5	19	2	6	60	1
Deep fried foods (Group 24)						
Potato fries	124	43.4	1	75	37.7	1
Sopaipilla	21	7.3	4	42	21.1	2
Fried empanadas	26	9.1	3	20	10.1	4
Spring rolls	8	2.8	7	4	2.0	7
Wontons	0	0	8	1	0.5	8
Chicken nuggets	21	7.3	5	27	13.6	3
Fried fish	44	15.4	2	17	8.5	5
Fried chicken ^c^	21	7.3	6	7	3.5	6
Others	21	7.3	-	6	3	-
Fluid milk (Group 25)						
Milk	871	67.7	1	733	70.2	1
Powdered milk	414	32.3	2	311	29.8	2
Sweet tea/coffee/milk drinks (Group 26)						
Coffee with sugar	69	7.1	2	2	0.2	3
Tea with sugar	472	48.4	1	141	15.9	1
Herbal tea with sugar	11	1.1	3	3	0.3	2
Mate tea with sugar	0	0	4	0	0	4
Flavored milk ^c^	423	43.4	-	742	83.6	-
Fruit juice (Group 27)						
Fruit juice	242	16.8	4	53	4.6	4
Packaged juice	268	18.6	3	488	42.3	1
Fruit drinks	619	43	1	487	42.2	2
Others	36	2.5	5	126	10.9	3
Soft drinks (Group 28)						
Soft drinks such as Coca-Cola, Fanta, or Sprite	684	98.7	1	445	99.1	1
Energy drinks such as Red Bull	1	0.1	4	0	0	3
Sports drinks such as Gatorade	8	1.2	3	4	0.9	2
Fast food (Group 29)						
McDonald’s	25	26.6	1	21	38.2	1
Burger King	10	10.6	2	0	0	5
KFC	9	9.6	3	6	10.9	2
Doggi’s	6	6.4	4	2	3.6	4
Pizza Hut	5	5.3	5	5	9.1	3
Others	39	41.5	-	21	38.2	-

^a^ Foods were classified according to the DQQ food groups and the Minimum Dietary Diversity for Women (MDD-W) guide 2021. ^b^ Number of times that food or beverage (>15 g) was reported in 24HRs. ^c^ Proposal corresponds to foods not described in the DQQ but that presented a high frequency in the sample (>4%). Others correspond to foods not described in the DQQ and had a low frequency in the sample (<3%). Group 1: doughs, fajitas, arepas, flour, cornstarch, semolina, couscous, and others; Group 2: whole wheat crackers and whole wheat pasta; Group 3: plantain and sweet potato; Group 4: fava beans and soy sprouts; Group 6: watercress and arugula; Group 7: green bell peppers, hearts of palm, chilis, brussels sprouts, eggplant, radish, and cochayuyo, among others; Group 8: papaya; Group 9: grapefruit; Group 10: honeydew melon, pomegranate, quince, pineapple, prickly pear, custard apple, and dried fruit, among others; Group 11: pancakes and sweet doughs; Group 12: canned fruit, spreads, syrups, and others; Group 16: organs and viscera; Group 17: industrialized hamburgers and organs and viscera; Group 21: cashews and pistachios; Group 24: hand rolls and onion rings; Group 26: flavored milk, milkshakes, and sweetened cocoa powder/flavored powder; Group 27: dairy drinks; Group 29: foods from national and international fast food chains.

**Table 3:** Diet quality indicators among pregnant women and children.

Indicator	Pregnant Women *n* = 1418	Children *n* = 799
GDR ^a^	9.3 (2.20)	8.1 (2.05)
NCD-Protect ^a^	2.5 (1.34)	1.7 (1.30)
NCD-Risk ^a^	2.3 (1.55)	2.6 (1.48)
MDD-W, % (*n*)	65.2 (925)	45.4 (363)
DDS ^a^	5.0 (1.31)	4.4 (1.34)
Zero vegetable or fruit consumption, % (*n*)	7.3 (104)	23.0 (184)
Protective Food Consumption, % (*n*)	36.7 (520)	22.6 (181)
More than one sugary food or beverage, % (*n*)	60.2 (853)	73.7 (589)
More than one salty ultra-processed food, % (*n*)	9.7 (138)	13.2 (106)

^a^ Values are given as mean (SD). GDR, Global Dietary Recommendations; NCD, noncommunicable disease; MDD-W, Minimum Dietary Diversity for Women; DDS, Dietary Diversity Score. Zero vegetable or fruit consumption is the proportion of the population that did not consume any vegetable or fruit; Protective Food Consumption is the proportion of the population that consumed at least one fruit, at least one vegetable, and at least one whole grain, legume, nut, or seed. More than one sugary food or beverage is the proportion of the population that consumed more than one sugary food or beverage in the previous day or night (e.g., baked or grain-based sweets, and other sweets; soft drinks, sweet tea/coffee/cocoa, and fruit drinks). More than one salty ultra-processed food is the proportion of the population that consumed more than one salty food in the previous day or night (e.g., processed meats, packaged ultra-processed salty snacks, instant noodles, deep fried foods, and fast food).

**Table 4:** Associations between food group-based diet quality scores, the consumption of NOVA food group, and total dietary energy intake ^a^.

Indicator	Pregnant Women			Children		
	% kcal NOVA Group 1	% kcal NOVA Group 4	% Total Dietary Energy Intake	% kcal NOVA Group 1	% kcal NOVA Group 4	% Total Dietary Energy Intake
GDR	0.3852 *	−0.307 *	−0.2438 *	0.3679 *	−0.387 *	−0.1101 *
NCD-Protect	0.2707 *	−0.1281 *	0.0364	0.2872 *	−0.2359 *	0.1425 *
NCD-Risk	−0.3236 *	0.3342 *	0.3872 *	−0.2649 *	0.3279 *	0.2915 *
DDS	0.2646 *	−0.1496 *	0.1355 *	0.3178 *	−0.2431 *	0.1898 *

^a^ Values are Spearman rank correlation coefficients. * Significant at *p* < 0.001. GDR, Global Dietary Recommendations; NCD, noncommunicable disease; DDS, Dietary Diversity Score. NOVA food classification system: Group 1, unprocessed or minimally processed foods; Group 4, ultra-processed foods.

## Data Availability

The original contributions presented in this study are included in the article. Further inquiries can be directed to the corresponding author.

## Supplementary Materials

The following supporting information can be downloaded at: https://www.mdpi.com/article/10.3390/nu17182980/s1, Supplementary File S1. DQQ Chile and suggested modifications to improve food groups DQQ in the Chilean version. Supplementary File S2. Guide Indicators calculated from food group DQQ.

## Abbreviations

The following abbreviations are used in this manuscript:

DQQ	Diet Quality Questionnaire
GDR	Global Dietary Recommendations
NCD	noncommunicable disease
MDD-W	Minimum Dietary Diversity for Women
DDS	Dietary Diversity Score
UPFs	ultra-processed foods
CIAPEC	Center for Research in Food Environments and Prevention of Nutrition-Related Chronic Diseases
