# Supplementary material for: Validating Sentinel Foods in the Diet Quality Questionnaire: Insights from Two Chilean Cohorts of Pregnant Women and Children
Source: Nutrients. 2025 Sep 17;17(18):2980. doi: 10.3390/nu17182980 (PMC12473036; doi:10.3390/nu17182980)
Supplement: Supplementary file 1 [file nutrients-17-02980-s001.zip › nutrients-3811923-Supplementary File S1.pdf]

Supplementary File S1

DIET QUALITY QUESTIONNAIRE (DQQ)

CHILE

READ: Now I'd like to ask you some yes-or-no questions about foods and drinks that you consumed yesterday during the day or night, whether you had it at home or somewhere else.

First, I would like you to think about yesterday, from the time you woke up through the night. Think to yourself about the first thing you ate or drank after you woke up in the morning … Think about where you were when you had any food or drink in the middle of the day … Think about where you were when you had any evening meal … and any food or drink you may have had in the evening or late-night … and any other snacks or drinks you may have had between meals throughout the day or night.

I am interested in whether you had the food items I will mention even if they were combined with other foods.

Please listen to the list of foods and drinks, and if you ate or drank ANY ONE OF THEM, say yes.

	**Yesterday, did you eat any of the following foods:**	**(circle answer)**
1	**White bread, pasta, or rice?**	YES or NO
2	**Oats, corn, wheat berries, whole grain bread, or quinoa?**	YES or NO
3	**Potato?**	YES or NO
4	**Beans, chickpeas, lentils, peas, soy meat, or hummus?**	YES or NO
	**Yesterday, did you eat any of the following vegetables:**	
5	**Carrots, zapallo squash, or red pepper?**	YES or NO
6	**Broccoli, chard, or spinach?**	YES or NO
7.1	**Tomatoes, lettuce, cucumber, green beans, cabbage, or cauliflower?**	YES or NO
7.2	**Zucchini, beet, celery, artichoke, asparagus, or mushrooms?**	YES or NO
	**Yesterday, did you eat any of the following fruits:**	
8	**Cantaloupe, apricots, mango, or loquat?**	YES or NO
9	**Orange or mandarin?**	YES or NO
10.1	**Banana, apple, pear, peaches, plums, kiwi, or watermelon?**	YES or NO
10.2	**Avocado, grapes, cherries, strawberries, raspberries, mora, or blueberries?**	YES or NO
	**Yesterday, did you eat any of the following sweets:**	
11	**Cookies, cakes, quick sweet breads, Chilean pastries, churros, calzones rotos, or donuts?**	YES or NO
12	**Candy, masticables, chocolates, ice cream or popsicle, or manjar?**	YES or NO
	**Yesterday, did you eat any of the following foods of animal origin:**	
13	**Eggs?**	YES or NO
14	**Cheese, hard cheese, or fresh cheese?**	YES or NO
15	**Yogurt or cultured milk?**	YES or NO
16	**Cold cuts and sausages such as ham, bologna, hot dogs, chorizo sausage, longaniza sausage, salami, or bacon?**	YES or NO
17	**Beef, beef liver, lamb, or goat?**	YES or NO
18	**Pork?**	YES or NO
19	**Chicken or turkey?**	YES or NO
20	**Fish, jurel, tuna, sardines, or seafood?**	YES or NO
	**Yesterday, did you eat any of the following other foods:**	
21	**Peanuts, peanut butter, almonds, walnuts, Chilean hazelnuts, Chilean pine nuts, or chestnuts?**	YES or NO
22	**Potato chips, Ramitas, Cheetos, Doritos, or Suflés?**	YES or NO
23	**Instant soup or instant noodles such as Maruchan?**	YES or NO
24	**Potato fries, sopaipilla, fried empanadas, spring rolls, wontons, chicken nuggets, or fried fish?**	YES or NO
	**Yesterday, did you have any of the following beverages:**	
25	**Milk or powdered milk?**	YES or NO

26	**Coffee with sugar, tea with sugar, herbal tea with sugar, or mate tea with sugar?**	YES or NO
27	**Fruit juice, packaged juice, or fruit drinks?**	YES or NO
28	**Soft drinks such as Coca-Cola, Fanta, or Sprite, energy drinks such as Red Bull, or sports drinks such as Gatorade?**	YES or NO
	**Yesterday, did you get food from any place like...**	
29	**McDonald's, Burger King, KFC, Doggi's, or Pizza Hut?**	YES or NO

Adapted by the Global Diet Quality Project, www.dietquality.org. 2021.
Supported by the EU and BMZ (through GIZ), USAID, The Rockefeller Foundation, and SDC.

INSTRUCTIONS:

1) Read the DQQ exactly as written. Do not include additional dialogue or probing questions. Do not add or remove food items. It is important for the integrity of the questionnaire and comparability of results to not modify the DQQ. Further instructions on how to use the DQQ can be found at our website at www.dietquality.org

2) If you desire to collect information on additional food items, or to add supplementary questions, these additional questions can be placed at the end of the questionnaire.

3) The Global Diet Quality Project has undertaken a systematic process to identify food items for each food group. If you would like to suggest changes to the DQQ, please contact the team using the “Contact Us” button on our website.

Table S1: Based on the analysis, the following modifications are suggested to improve the capture of food groups of the DQQ in the Chilean version:

DQQ Food Group	Original sentinel	Proposal	Motive
11. Baked / grain-based sweets	11. Cookies, cakes, quick sweet breads, Chilean pastries, churros, calzones rotos, or donuts?	Add cereal bars<br>Remove churros	A low consumption of churros and calzones rotos (n=28) was observed among pregnant women and children, in contrast to the high consumption of cereal bars (n=104). The latter is high in sugar in the Chilean context.
12. Other sweets	12. Candy, masticables, chocolates, ice cream or popsicle, or manjar?	Add milk-based dessert and jellies	A high consumption of milk-based desserts and jellies was observed in both cohorts. (n=267)
16. Processed meats	16. Cold cuts and sausages such as ham, bologna, hot dogs, chorizo sausage, longaniza sausage, salami, or bacon?	Add patés/spreads<br>Remove bacon	We observed high consumption of products such as patés and spreads (n=82), while bacon had low consumption (n=5)
22. Packaged ultra-processed salty snacks	22. Potato chips, Ramitas, Cheetos, Doritos, or Suflés?	Add salty crackers	We observed high consumption of saltine crackers, with a rate higher than that reported for sentinel foods (n=139). In Chile, salty crackers are often consumed as chips.
24. Deep fried foods	24. Potato fries, sopaipilla, fried empanadas, spring rolls, wontons, chicken nuggets, or fried fish?	Add fried chicken<br>Redacting nuggets<br>Exclude wonton	Remove wontons from the list, as they are not commonly consumed. Change "nuggets de pollo" to simply "nuggets," which can encompass both fish and chicken nuggets. Additionally, include fried chicken, as it is a commonly consumed item.
26. Sweet tea/coffee/milk drinks	26. Coffee with sugar, tea with sugar, herbal tea with sugar, or mate tea with sugar.	Add flavored milk with added sugar or regular cocoa powder.	"High consumption of flavored milk (n = 1,165) was observed in both cohorts; however, after the law was enacted, many reformulated products incorporated non-caloric sweeteners. The next step is to identify only those flavored milk products that contain added sugar or regular cocoa powder."
