# Supplementary material for: Validating Sentinel Foods in the Diet Quality Questionnaire: Insights from Two Chilean Cohorts of Pregnant Women and Children
Source: Nutrients. 2025 Sep 17;17(18):2980. doi: 10.3390/nu17182980 (PMC12473036; doi:10.3390/nu17182980)
Supplement: Supplementary file 1 [file nutrients-17-02980-s001.zip › nutrients-3811923-Supplementary File S2.pdf]

Supplementary File S2: Guide to calculating indicators from DQQ.

Dietary Diversity Score (DDS)	DQQ Question numbers	Points
Grains, white roots and tubers, and plantains	1, 2, 3	1
Pulses (beans, peas and lentils)	4	1
Nuts and seeds	21	1
Dairy	14, 15, 25	1
Meat, poultry and fish	16, 17, 18, 19, 20	1
Eggs	13	1
Dark green leafy vegetables	6	1
Other vitamin A-rich fruits and vegetables	5, 8	1
Other vegetables	7.1, 7.2	1
Other fruits	9, 10.1, 10.2	1
		SUM=0-10

Minimum Dietary Diversity (MDD)	
If score from DSS ≥ 5	1
If score from DSS <5	0

NCD-Protect Score	DQQ Question numbers	Points
Whole grains	2	1
Pulses	4	1
Nuts and seeds	21	1
Other vitamin A-rich vegetables	5	1
Dark green leafy vegetables	6	1
Other vegetables	7.1, 7.2	1
Other vitamin A-rich fruits	8	1
Citrus	9	1
Other fruits	10.1, 10.2	1
		SUM=0-9

NCD-Risk Score	DQQ Question numbers	Points
Soft drinks	28	1
Baked / grain-based sweets	11	1
Other sweets	12	1
Processed meat	16	2
Unprocessed red meat	17, 18	1
Deep fried food	24	1
Fast food & Instant noodles	23, 29	1
Packaged ultra-processed salty snacks	22	1
		SUM=0-9

The **GDR score** is calculated as follows: NCD-Protect - NCD-Risk + 9 = GDR score
